# Protein assembly systems in natural and synthetic biology

**DOI:** 10.1186/s12915-020-0751-4

**Published:** 2020-03-26

**Authors:** Giulio Chiesa, Szilvia Kiriakov, Ahmad S. Khalil

**Affiliations:** 1 Biological Design Center, Boston University, Boston, MA 02215 USA; 2 Department of Biomedical Engineering, Boston University, Boston, MA 02215 USA; 3 Wyss Institute for Biologically Inspired Engineering, Harvard University, Boston, MA 02115 USA

## Abstract

The traditional view of protein aggregation as being strictly disease-related has been challenged by many examples of cellular aggregates that regulate beneficial biological functions. When coupled with the emerging view that many regulatory proteins undergo phase separation to form dynamic cellular compartments, it has become clear that supramolecular assembly plays wide-ranging and critical roles in cellular regulation. This presents opportunities to develop new tools to probe and illuminate this biology, and to harness the unique properties of these self-assembling systems for synthetic biology for the purposeful manipulation of biological function.

## Introduction

Proteins are the workhorse molecules of the cell, driving virtually every function and developmental program in biology. Amazingly, many of these critical molecules readily aggregate and assemble inside of living cells through interactions amongst unfolded and folded domains. This can occur aberrantly and lead to disease; but there is also accumulating evidence that aggregation phenomena can be regulated by the cell and used to carry out important and beneficial biological functions ranging from molecular scaffolding to memory [1–4]. Moreover, when designing synthetic cellular systems using synthetic biology, we argue that protein aggregation may be viewed as a “feature” rather than a “bug”, and that self-assembling elements possess unique properties that can be exploited to engineer new biological functions [5]. In this Review, we provide a brief introduction to protein assembly and the spectrum of aggregation phenomena found in nature, we survey the diverse and rapidly expanding set of biological functions driven by supramolecular assemblies, and finally we offer a prospective discussion of the methods and benefits of their purposeful manipulation in cells and organisms.

## Biological parts

**Fig. 1:**
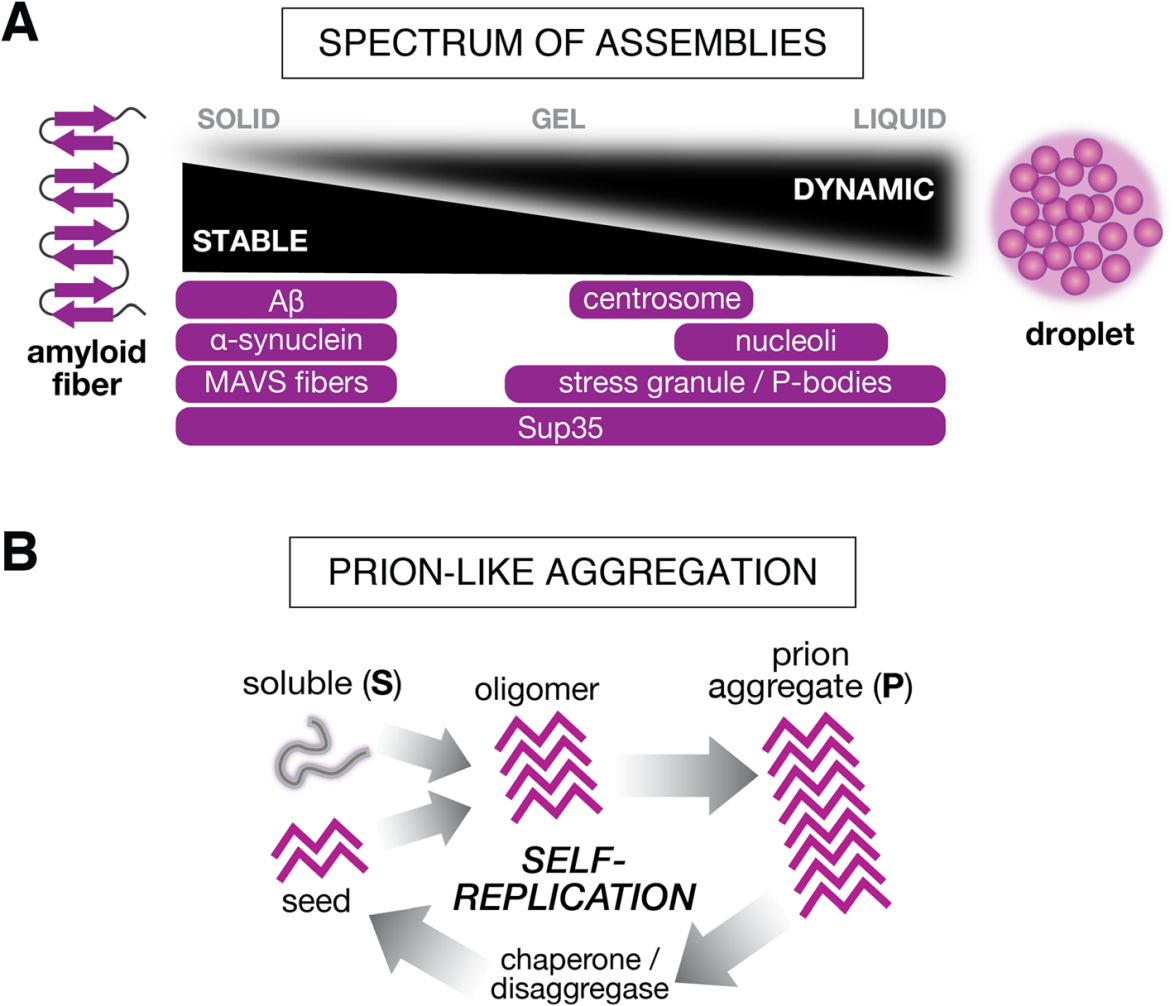
Overview of higher-order assemblies. **a** Protein assemblies display a spectrum of material properties, from solid-like amyloid fibers to highly dynamic liquid droplets. Examples of assemblies are shown below the spectrum. Highly stable assemblies include MAVS (mitochondrial antiviral signaling) protein fibers and Aβ (amyloid β) peptide amyloid fibrils. Highly dynamic assemblies include nucleoli, membraneless organelles with liquid-like shell around a more organized rigid core. The yeast prion protein Sup35 can convert between different structures: it constructs stable amyloid fibrils in its prion conformation and undergoes reversible gel formation under pH stress. Stress granules and P-bodies can also exist in different states, depending on the physiology of the cell. **b** Prions are self-propagating protein conformations. The prion conformation (*purple*) serves as a template to convert the soluble (*gray*) conformation into the prion conformation, which usually results in the growth of amyloid aggregates. The aggregates are fragmented by chaperone proteins, producing seeds that can nucleate the conformational conversion

Protein components can self-assemble into higher-order complexes or assemblies within the cell. A common feature of many of these proteins is the presence of intrinsically disordered regions (IDRs). IDRs are protein sequences that do not adopt a single three-dimensional structure, but instead endow proteins with flexibility to adopt a range of states, from unstructured to partially structured [6]. Due to this flexibility, IDRs can enable proteins to engage multiple partners and participate in the different types of interactions that facilitate initiation of protein assembly, e.g., (1) specific interactions among or between folded domains and unfolded sequences [7–9] and (2) non-specific weak interactions among IDRs [10, 11]. Depending on the relative strength and avidity of these interactions, as well as other factors such as the physical-chemical state of the cellular environment, a broad spectrum of assembly phenomena can arise (Fig. 1). On one end of the spectrum, proteins can be recruited and maintained in highly dynamic, metastable assemblies that are characterized by liquid-like properties [12, 13]; at the other end of the spectrum, these initial interactions can give rise to more ordered interactions that produce stable higher-order aggregates, like amyloid fibers. Below, we provide a brief overview of these different classes of supramolecular assemblies, discussing their key properties and hallmark examples.

### Dynamic assemblies

At the dynamic end of the spectrum are supramolecular assemblies based on phase separation. Biological phase separation has generated tremendous excitement as recent discoveries point to its critical role in many cellular processes, such as the formation of organelles and regulatory complexes, as well as in human diseases [14]. Our goal is to provide the reader a very brief introduction and primer to this phenomenon; for more details on the properties, formation, regulation, and function of this class of assembly, we refer the reader to several recent reviews [12, 14–16]. For simplicity, we will broadly refer to these assemblies as dynamic condensates or dynamic assemblies [17].

Phase separation is a physical phenomenon that occurs when a solution of molecular components spontaneously separates or demixes into co-existing (lower free energy) phases, such as when oil is mixed with water [1]. In cells, molecular assembly via phase separation provides a general strategy for organizing and compartmentalizing biological matter. Specifically, protein-based phase separation can drive the formation of cellular compartments and organelles that lack membrane boundaries. A canonical example is the nucleolus, a large and dynamic complex of proteins and RNA found within eukaryotic nuclei that is the site of ribosome biogenesis. Nucleoli can display a broad range of sizes, coalesce into larger droplets, and display a range of viscosities, all of which represent classic properties of liquids [18]. Nucleoli are just one example of a larger family of membraneless organelles. The first membraneless organelle characterized as liquid-like was the P-granule [19]. Since then, P bodies, stress granules, Cajal bodies, nuclear speckles, PML bodies, and germ granules have all been demonstrated to form through phase separation and possess liquid-like physical properties [20, 21].

As discussed more below, these dynamic assemblies can help to compartmentalize biological reactions and in some cases can even be composed of distinct, non-miscible sub-compartments with different protein compositions [22]. In general, weak multivalent interactions among components of the assembly drive the formation of condensates and regulate their dynamic properties. The interactions can be summarized into two main types: (1) specific interactions among folded domains [23] or between folded domains and unfolded sequences [7–9, 24]; and (2) non-specific weak interactions among IDRs [10, 11]. In the first case, the strength and valency of interactions and the relative component concentrations determine their phase behavior, which can span reversible droplet formation to gelation into a meshwork of non-covalently crosslinked hydrogels [8]. Proteins that undergo the second type of interaction often have disordered regions with specific sequence compositions, called low complexity regions (LCRs). LCRs feature highly repetitive sequences, such as polyglutamine (polyQ) repeats or glutamine/asparagine (Q/N)-rich motifs. Other key elements of these regions are clusters of hydrophobic and aromatic residues [25] and patches of charged side chains [26]. Interestingly, the dynamics of phase transition and the physical properties of the assembly, such as the viscosity, are intimately related to the sequence composition of the constituent proteins [22, 27], presenting unique opportunities to elucidate a “molecular grammar” [27] for assembly formation and to create designer assemblies based on sequence alone.

### Stable assemblies: amyloid fibers

At the other end of the spectrum are solid structures, such as amyloid fibers. Amyloids are highly stable assemblies composed of protein molecules organized in a cross-β-sheet lattice of indefinite length [28]. Amyloid fibrils form through an unfolded-to-folded transition, wherein partially unfolded protein sequences lock into β-sheet conformations and self-assemble by aligning their β-sheets and subsequently growing in a linear fashion [29]. This process is generally described as nucleation-dependent polymerization, where the protein needs to access a metastable conformation in order to trigger aggregation. Once this state is achieved, the reaction proceeds rapidly to completion as a first-order reaction [30–33].

Proteins with high propensity to form amyloids contain disordered regions enriched in hydrophobic and polar amino acid residues. However, amino acid composition alone is insufficient to predict amyloid-forming capacity, as the position within the protein sequence also matters. Collectively, these insights have been used to predict amyloid-forming sequences in the proteomes of organisms [34, 35].

Amyloid fibrils are extraordinarily robust biomaterials. They are protease-, heat-, and detergent-resistant and can have stiffness comparable to that of spider silk and collagen fibers [36]. Both protein sequence and the physical/chemical conditions of the solvent [37, 38] contribute to the biophysical properties of the resulting fibril, such that the same underlying protein can form fibrils with a variety of morphologies and degrees of rigidity [38].

Amyloids have been linked to many human diseases, ranging from diabetes to systemic amyloidoses [39, 40]. Perhaps most notably, amyloid deposits are hallmarks of neurodegenerative disorders and their formation has been linked to the etiology of diseases, such as Alzheimer’s disease (AD) and Parkinson’s disease (PD) [41]. However, as described below, examples of non-disease, functional amyloids are being increasingly uncovered and characterized. For a more detailed treatment of these structures and their roles in disease and physiology, we refer the reader to other reviews [29, 42–44].

### Heritable assemblies: prions

Prions are a unique class of aggregating proteins. Prion proteins, first discovered as the causative agents of transmissible neurodegenerative disorders in mammals (e.g., scrapie, bovine spongiform encephalopathy, and Creutzfeld-Jakob disease [45, 46]), have several unusual properties. First, they can exist in functionally distinct conformational states. Conversion between a soluble conformation (associated with “normal” protein activity) and a prion conformation results in dramatic change in protein activity that can lead to new cellular phenotypes, including disease. Second, the prion conformation is self-replicating. That is, it serves as a template to convert the soluble conformation into the prion conformation; once induced, this prion state can self-replicate on long biological timescales [47, 48]. Third, the prion conformation is infectious: it can be transmitted from one cell to another and, in the case of bona fide prions such as the disease-causing human prion protein (PrP), from one organism to another. Because of these properties, intracellular prions and their associated phenotypes are heritable [42]. Proteins that fulfill all of these criteria except for inter-organismal transmission are often referred to as prionoids, and there is accumulating evidence that proteins associated with human neurodegenerative diseases, such as Tau in AD, have prion-like seeding and spreading properties [49].

Most known prions form amyloid aggregates, templating the amyloid conformation onto newly synthesized proteins. Conversion between the prion and soluble conformation is a reversible process [50]. However, once insoluble amyloid fibers are formed, they are generally considered irreversible: amyloids are not cleared by the protein quality control system and stored as inclusions in cellular compartments [51, 52]. Not all prions are known to form amyloids. Certain prion proteins can also form dynamic condensates [32, 53]; others have yet undetermined conformations [54], and it is likely these elements can take on a broad spectrum of biophysical and conformational properties.

Much of our molecular understanding of prions originates from studies in yeast, where over ten bona fide prion proteins have been characterized to date (Table 1) [60, 73, 82]. Unlike mammalian PrP, certain prions in yeast have been proposed to play useful regulatory functions (see below). These studies have revealed two common molecular properties. First, canonical prions contain prion-forming domains (PrDs), which are modular intrinsically disordered domains (often Q/N-rich) that confer their prionogenic behavior. In silico screens trained on known PrDs have been able to predict new prion sequences [68, 83–86]. However, it should be noted that not all known prions have PrDs and canonical sequence properties, and thus identification exclusively on these requirements is insufficient [66]. Second, prions interact with and depend on the activity of chaperone proteins for propagation. In yeast, transmission and maintenance of the prion state are highly dependent on the disaggregase Hsp104, which serves to fragment amyloid fibers and create infectious, low molecular weight prion “seeds” that infect daughter cells [87]. Though recent studies suggest that *Saccharomyces cerevisiae* may harbor many more uncharacterized prionoid elements that appear to be independent of Hsp104, and instead dependent on other chaperones [81, 88]. Overall, new experimental tools will be needed to discover and characterize these elements.

**Table 1:** Prion proteins: bona fide prions, prion candidates, and prionoids

Prion	Protein determinant	Function	Prion phenotype	Organism	Prion properties	Reference
Amyloid A		Apolipoprotein, inflammatory response	Amyloid A amyloidosis	Human, cattle, cheetah, chicken, mouse	Amyloid, cell-to-cell spreading, trans-organismal spreading (except for humans)	[43]
[β]	Prb1	Vacuolar proteinase B, protein degradation in vacuole	Phenotypic lag: prolonged carboxypeptidase Y activity after loss of *PEP4*	*Saccharomyces cerevisiae*	Non-amyloid, reversibly curable, mitotic inheritance, infectious	[55]
CPEB, Orb2, CPEB3*		Regulating synaptic plasticity, repressor of AMPA receptor transcription	Facilitating long-term memory, activator of AMPA receptor translation	Sea slug, fruit fly, mouse	Self-templating, amyloid, heritable in yeast	[56–58]
Cb-Rho prion*	Rho	Transcriptional global regulator	Transcriptional terminator read-through	*Clostridium botulinum*, *Escherichia coli*	Amyloid, Sup35C assay, inheritance	[59]
[*GAR*^+^]	Pma1/Std1	Proton pump, glucose signaling	Utilization of poor carbon sources in the presence of glucose	*Saccharomyces cerevisiae*	Inheritance, infectious	[54, 60]
[*Het-s*]	Het-s	Heterokaryon incompatibility upon mixing with self		*Podospora anserina*	Dependence on Pa Hsp104, amyloid fibrils infectious	[61, 62]
[*LD*^+^]*	Lumini-dependens	Transcription factor controlling autonomous flowering pathway	Hypothetical: delay of flowering	*Arabidopsis thaliana*	Sup35C assay, detergent resistant aggregates, Hsp70 and Hsp90 dependence, inheritance, infectious	[63, 64]
[*LSB*^*+*^]	Lsb2	Cytoskeletal assembly protein	Seeds [*PSI*^*+*^]	*Saccharomyces cerevisiae*	Dependence on Hsp104, inheritance, infectious, detergent resistant aggregate	[65]
[*MOD*^+^]	Mod5	tRNA isopentenyltransferase	Resistance to azole antifungals, modified sterol biosynthesis	*Saccharomyces cerevisiae*	Amyloid, Hsp104 dependence, inheritance, infectious	[66, 67]
[*MOT3*]	Mot3	Transcriptional regulation	Increased biofilm formation, agar invasion, decreased hypoxia resistance	*Saccharomyces cerevisiae*	Amyloid, dependence on Hsp104, inheritance, infectious	[60, 68]
[*NSI*^+^]	Unknown	Unknown	Translational read-through affecting the Sup45 termination factor	*Saccharomyces cerevisiae*	Sup35C assay, dependence on Hsp104, inheritance, infectious	[60, 69]
[*NUP100*^+^]	Nup100	Nuclear pore complex	No significant effect	*Saccharomyces cerevisiae*	Amyloid, self-assembly, Sup35C assay, dependence on Hsp104, mitotic inheritance	[67, 70, 71]
[*OCT*^+^]	Cyc8	Ttranscriptional regulation, chromatin regulation	Derepression of genes inhibited by Cyc8-Tup1 complex, invertase production in the presence of glucose, flocculation	*Saccharomyces cerevisiae*	Dependence on Hsp104, inheritance, infectious	[60, 72]
[*PSI*^+^]	Sup35	Translational termination	Translational read-through	*Saccharomyces cerevisiae*	Amyloid, Sup35C assay, dependence on Hsp104, inheritance, infectious	[60, 67, 73, 74]
PrP Sc	PRNP	Preserves synaptic structure and function	Fatal neurodegenerative diseases	Human, sheep, cattle, deer, mink, felines	Self-templating, detergent and protease resistant amyloid fibers, infectious	[75]
[*RNQ*^*+*^]	Rnq1	Unknown	Cross-seeding other prions	*Saccharomyces cerevisiae*	Amyloid, Sup35C assay, dependence on Hsp104, inheritance, infectious	[60, 67, 73, 74]
[*SWI*^+^]	Swi1	Transcriptional regulation, nucleosome remodeling	Poor growth on non-glucose carbon sources, abolished multicellular features	*Saccharomyces cerevisiae*	Amyloid, Sup35C assay, dependence on Hsp104, inheritance, infectious	[60, 67, 73, 74]
[*URE3*]	Ure2	Gln3 repressor	Utilization of poor nitrogen sources in the presence of ammonium	*Saccharomyces cerevisiae*	Amyloid, Sup35C assay, inheritance, infectious	[60, 67, 73, 74]
Prionoid	Protein determinant	Function	Prion phenotype	Organism	Prionoid properties	Reference
ASC		Adaptor protein for inflammasome signaling	Inflammasome activation	Human	Non-amyloid helical polymer, cell-to-cell spreading PYD domain in yeast: Sup35C assay, detergent resistant aggregates, inheritance	[76, 77]
[*LEF*^+^]	LEF-10	Viral late expression factor	Downregulation of viral late gene expression	*Autographa californica* multiple nucleopolyhedrovirus	Sup35C assay, detergent resistant aggregates	[78]
MAVS*		Signal transduction from mitochondrial membrane to cytosol	Activation of MAVS, initiation of antiviral signaling	Human, mouse	Self-templating, detergent and protease-resistant fibers	[79]
[*NU*^+^]	New1 PrD	ATP binding cassette protein	Susceptibility to [*PSI*^+^] induction	*Saccharomyces cerevisiae*	Sup35C assay, amyloid, full New1 protein not shown to form prions	[74, 80] [68]
[*NRP*^+^]	Nrp1**	Putative RNA binding protein		*Saccharomyces cerevisiae*	Sup35C assay, detergent-resistant aggregates, Hsp104 dependence	[68, 74]
p53		Transcriptional regulator	Metastasis	Human, mouse	Large cytoplasmic inclusions, cell-to-cell spreading	[43]
Islet amyloid polypeptide (IAPP)		Glycemic regulation	Type 2 diabetes	Human, mouse	Cell-to-cell spreading, self-templating amyloid	[43]
**Prion candidates**					**Organism**	**Reference**
Amyloid A, amyloid-β, −synuclein, β2-microglobulin, immunoglobulin light chain, tau, transthyretin					Human	[43]
ASM4, CBK1, GLN3, GPR1, GTS1, HRP1, KSP1, LSM4, NGR1, NRP1, NSP1, PDR1, PGD1, PUB1, PUF2, RBS1, RLM1, SAP30, YBL081W, YBR016W, YPL184C, YPR022C					*Saccharomyces cerevisiae*	[68]
ASH1, AZF1, BUD2, CSR2, ERG11, FRE1, GMC1, HAA1, HAP4, HEH2, HRD3, ILV1, JNM1, KAP120, KAP95, MGA1, MPH1, MRN1, MRPL10, MRS3, PBP2, PCL9, PIB1, POL32, PSP1, PUS4, RBS1, RLM1, SAP1, SBE2, SCD5, SED5, SEN15, SLI15, SMP1, SNT1, SPC110, STE20, STE5, UBX7, ULP1, VTS1, YCK3, YGL036W, YFH1, YLR152C					*Saccharomyces cerevisiae*	[81]

*Validation in non-native host organism. **Incomplete validation. In the prion nomenclature, brackets denote non-Mendelian inheritance and capital letters denote dominance in crosses. Prion properties include: amyloid—prion was shown to form amyloid or detergent resistant aggregates; inheritance—prion is inherited mitotically, meiotically, or as a fusion protein (Sup35C assay); chaperone dependence—prion is dependent on particular chaperones for propagation. Prion candidates are proteins that cannot officially be classified as bona fide prions because they lack experimental validation, but have the potential for inter-organismal spreading

Finally, some prions can assemble into multiple distinct self-templating structures. These structural variants—or conformational alleles—give rise to distinct and stable prion activities, called strains [89–92]. The concept of the “prion strain” was first suggested based on studies of PrP, in which different structures of the prion protein were identified and linked to distinct disease pathologies [93]. Now, many prions are known to give rise to strains, and it has been shown that strain variation derives from different amyloid fiber structures (with different physical properties) that correlate with heritable phenotypes [37, 94, 95]. For example, a number of strains have been identified for the well-studied yeast prion [*PSI*^+^], which is formed by self-templated aggregation of the translation termination factor Sup35 [96]. Two of the most common [*PSI*^+^] strains are referred to as weak and strong [91]. Strains carrying weak [*PSI*^+^] have lower efficiency of translational read-through relative to strong [*PSI*^+^], and thus a weaker phenotype. However, weak [*PSI*^+^] fibers are thermodynamically more stable, with larger and more rigid amyloid cores compared to strong [*PSI*^+^]. This structural difference results in less efficient fragmentation by the chaperone machinery, and consequently the generation of fewer seeds, slower templating of new protein to the prion form, and less stable inheritance [97]. Understanding these sequence–structure–phenotype relationships will provide important blueprints to guide the purposeful manipulation and engineering of these powerful elements of inheritance.

## Biological functions

**Fig. 2:**
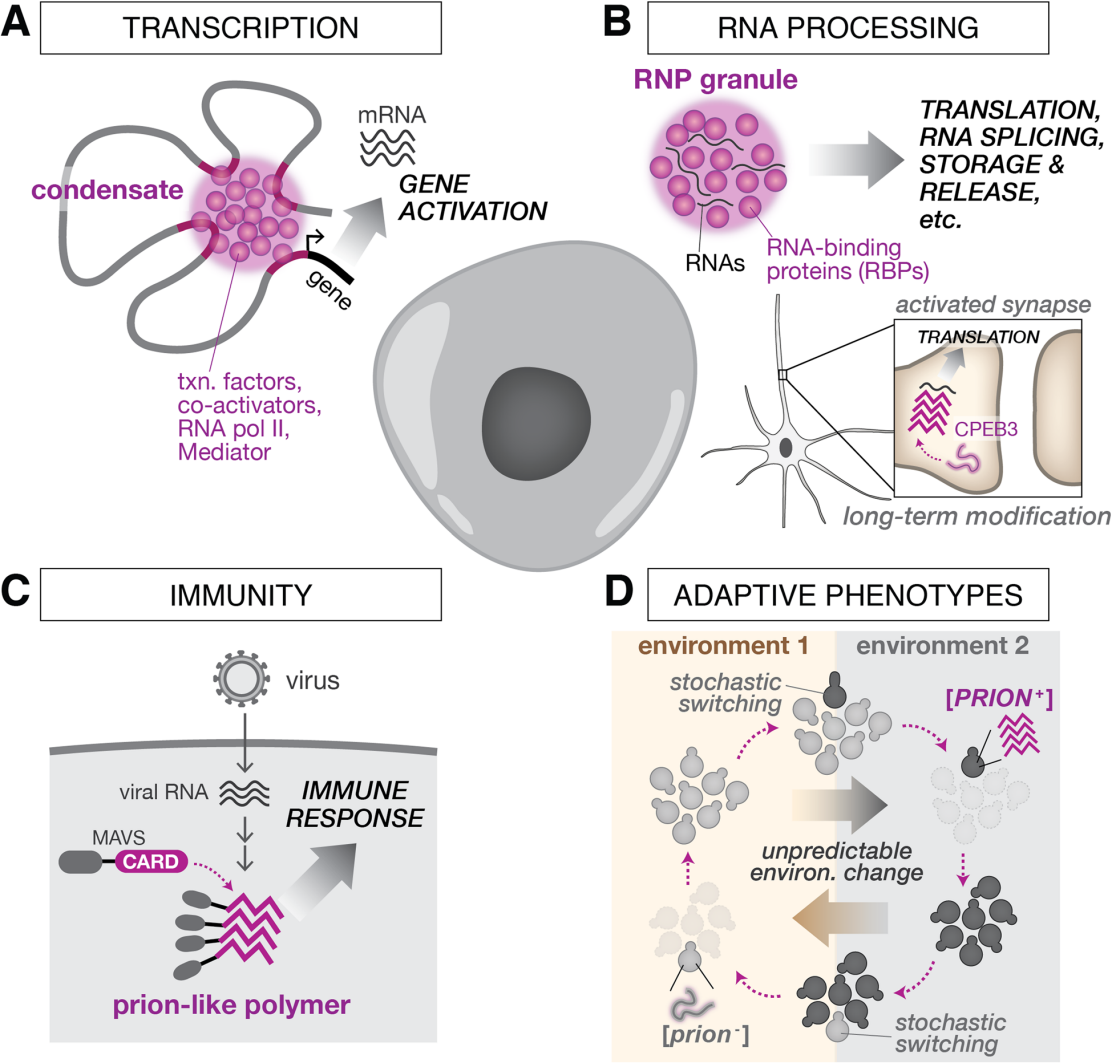
Protein assemblies play important roles in a variety of critical cellular processes. **a** In eukaryotic transcription, co-activators and transcription (*txn.*) factors form highly dynamic protein condensates that recruit RNA polymerase II (*RNA pol II*) and drive robust gene activation. **b** RNA-binding proteins (*RBPs*) and RNAs coalesce to form RNP granules, which serve different RNA processing functions, such as mRNA storage and degradation, ribosome biogenesis, and localized translation. In one intriguing example, prion-like aggregation of CPEB3 promotes translation in activated synapses to potentiate long-term memory. **c** Higher-order assemblies play key roles in innate immunity. For example, prion-like polymerization of the MAVS adaptor protein in response to viral infection leads to amplification and stabilization of the antiviral response. **d** In yeast, stochastic switching between [*prion*^−^] and [*PRION*^+^] states in a population of cells enables phenotypic diversification and may promote survival in uncertain environments. Figure adapted from Fig. 1B in [136]. In prion nomenclature, *brackets* denote non-Mendalian inheritance and *capital letters* denote dominance in crosses

Protein aggregation has been classically viewed as an aberrant process with pathological consequences. Indeed, cellular aggregates are associated with a large number of human diseases, including neurodegeneration [98], type 2 diabetes [39], and aging [99], and extensive work has been dedicated to elucidating their role in these and other diseases (reviewed elsewhere [42, 43]). But in part due to the development of new techniques to study protein aggregates (Table 2), we are gaining a new appreciation and understanding of their non-pathological roles. Protein aggregates play positive functions in a variety of cellular processes, including gene regulation [126, 127], signaling [76, 128, 129], memory storage [56, 130, 131], DNA repair [132], cell fate decisions [130, 133–135], and even evolution [2]. These examples should serve as inspiration for synthetic biologists aiming to purposefully manipulate information flow in living systems (Fig. 2).

**Table 2:** Methods to evaluate and quantify protein assemblies

In vitro:	
• The canonical approach to determine the amyloid nature of a protein aggregate is by staining with dyes, such as Thioflavin-T (ThT) and Congo Red, which selectively intercalate into the fibril and emit fluorescence at specific wavelengths in a quantitative fashion [100]. ThT-based assays can measure kinetics of aggregation in high throughput [28, 101, 102].	
• Semi-denaturing detergent agarose gel electrophoresis (SDD-AGE) characterizes the size distribution of large, detergent-resistant aggregates in cell lysates [103].	
• X-ray diffraction identifies the symmetry patterns of amyloid fibrils [104].	
• Solid-state nuclear magnetic resonance (ssNMR) is used to derive the structural properties of fibrils [105–107]. Solution NMR is used to elucidate the early stage dynamics of aggregate formation [108, 109], and the dynamics of conformational changes and interactions with other proteins [110].	
• Super-resolution microscopy techniques, such as PALM and STORM, have revealed the morphology of fibers [111] and cryo-electron microscopy has recently produced high-resolution images of protein aggregates [112, 113], all in fixed cells.	
In vivo:	
• Dynamic properties of protein assemblies can be studied with microscopy techniques, such as fluorescence recovery after photobleaching (FRAP) [114] and Forster resonance energy transfer (FRET) [32].	
• DAmFRET enables the determination of nucleation barriers of protein assemblies in living cells [32].	
• Super-resolution microscopy (PALM and STORM) has been applied to living cells to measure the growth of amyloid fibrils [115, 116] and visualize the nucleation process [33].	
• yTRAP (yeast Transcriptional Reporting of Aggregating Protein) is a genetic system enabling high-throughput sensing and control of protein aggregation states in yeast cells [117].	
• A generic sensor of protein aggregation in mammalian cells uses a fusion of HSP27 and GFP [118].	
• Split protein systems, such as split TetR in mammalian cells [119] and a tripartite β-lactamase in *Escherichia coli* [120], enable detection of the solubility of a specific protein.	
• Various phenotypic assays have been developed to detect prions by linking prion state with a growth phenotype or reporter in yeast [54, 68, 72, 121–124].	
• Transmissibility of bona fide prions is evaluated using cytoduction in yeast [125].

### Gene regulation

Many components controlling aspects of gene expression form dynamic protein assemblies that contribute to their regulatory mechanism. Strikingly, different steps of eukaryotic gene transcription appear to utilize regulated phase separation mechanisms [137]. A first step in transcription is the binding of transcription factors (TFs) to enhancer regions. Phase separation was found to be important in this process at super-enhancers, which are clusters of enhancers driving robust transcription of cell identity genes. In particular, certain TFs were shown to phase separate via their IDRs into liquid-like condensates that help to compartmentalize the transcriptional apparatus [138]. A following step in transcription involves Mediator, a complex that connects signals from TFs to RNA polymerase (Pol) II. Mediator has been shown to form phase-separated clusters both with TFs [127] and with Pol II [126] at active sites of transcription. Finally, the process of transcription elongation relies on phosphorylation of the C-terminal domain (CTD) of Pol II. This is accomplished in part by the enzyme complex positive transcription elongation factor b (P-TEFb). To ensure hyper-phosphorylation of the CTD and efficient elongation, P-TEFb undergoes phase separation into nuclear speckles capable of recruiting Pol II [139]. Interestingly, protein phase separation has also been implicated in gene silencing through recent work demonstrating that HP1α proteins, key factors involved in the formation of heterochromatin domains, have the ability to form liquid droplets in a regulated fashion [140–142].

Proteins regulating the RNA life cycle, downstream of transcription, are among the most prominent examples of molecules that undergo phase separation. RNA-binding proteins (RBPs) are particularly rich in disordered, low-complexity sequences. Many RBPs possess IDRs that have been shown to undergo liquid–liquid phase transition in cells [20, 22, 23], thus driving the formation of membraneless organelles important in RNA metabolism (these include nucleoli, stress granules, P-bodies, and Cajal bodies) [143]. As one example, condensation of components of the human miRISC complex facilitates recruitment of deadenylation factors that promote degradation and silencing of mRNAs [144]. RBPs have gained recent attention because, on the one hand, their aggregation can drive the formation of these functional membraneless RNP bodies, yet on the other hand, mutations in their low-complexity sequences are causal factors in neurodegenerative diseases, including amyotrophic lateral sclerosis (ALS) and multisystem proteinopathy (MSP) [145]. Interestingly, nucleotide repeat expansions, one class of mutation associated with neurotoxicity, which can cause gain or loss of function of genes encoding RBPs, have also been shown to alter the properties of RNAs. Specifically, repeat-containing RNAs have been shown to form gels in vitro by creating opportunities for multivalent base-pairing, and can accumulate in aberrant and potentially toxic nuclear foci in cells that sequester RBPs [146].

### Immunity

The innate immune system is an ancient and rapid first-line defense that higher organisms deploy to defend against invading pathogens. This system consists of interconnected signaling pathways that activate inflammatory responses in an effort to eliminate the pathogen, as well as regulate different types of cell death, such as apoptosis and necroptosis (programmed necrosis). These pathways must be able to be rapidly deployed, but also tightly controlled and balanced in order to prevent excessive inflammatory responses or cell death. Many signaling components involved in these pathways are capable of oligomerization to form higher-order assemblies, sometimes generically referred to as signalosomes [147]. This capacity for oligomerization is an important mechanism for increasing specificity of and amplifying signal transduction [76].

One prominent example is the RIP1/RIP3 necrosome mediating programmed necrosis, a form of cell death (distinct from apoptosis) that represents an important host defense mechanism [128]. Here, the RIP1 and RIP3 kinases form a functional amyloid-based signaling complex to trigger programmed necrosis. Importantly, RIP1 and RIP3 kinase activation is required for this amyloid complex formation, which in turn can further enhance kinase activation through phosphorylation, thereby amplifying/propagating the pronecrotic signal.

Another key host defense mechanism is activation of inflammatory responses. This is carried out by the inflammasome complex, which translates pathogen and cellular danger signals recognized by sensors, such as NLRP3, into inflammatory responses through the adaptor protein ASC. Intriguingly, ASC was shown to be a bona fide prion in yeast [76, 77, 129]. Thus, in response to upstream sensors, initial oligomerization of ASC can result in prion-like nucleation; this in turn enables the templating of other ASC molecules to form large polymers capable of robustly recruiting caspase-1 molecules to induce their activation and propagate the inflammatory signal. Similarly, viral infection triggers the prion-like aggregation of the mitochondrial antiviral-signaling (MAVS) adaptor protein into fibrillar structures, which in turn recruit other soluble MAVS proteins, amplifying and stabilizing the antiviral response [76, 148]. These examples highlight how prion-like polymerization may provide a (evolutionarily conserved) mechanism for highly sensitive and robust response to cellular signals.

### Memory

One of the most fundamental and remarkable aspects of organismal behavior is the ability to make memories of past events, and to subsequently modify behavior by learning. Cells have multiple mechanisms for making molecular memories that outlast the half-life of proteins. One mechanism is prion-like aggregation [130, 149]. In animals, the cytoplasmic polyadenylation element binding protein 3 is a highly conserved RBP (CPEB in *Aplysia*, Orb2 in *Drosophila*, and CPEB3 in mice) that plays a role in the formation of new memories [56, 150, 151]. Specifically, prion-like aggregation of CPEB3 in the synapses of stimulated neurons leads to the formation of RNA granules that bind and drive translation of mRNAs involved in synaptic plasticity and growth [57]. CPEB3, and possibly also other RBPs, represents a fascinating example of how conformational changes at the molecular scale can produce macroscopic changes in animal behavior, linking molecular self-replication, cellular memory, and neuronal memory.

### Evolution

Since their initial discovery, we have come to understand prions not only as causative agents of disease, but also as sources of new and sometimes adaptive cellular functions [47, 66, 88, 121, 136, 152–157]. This has been most apparent in yeast, where several central regulators of information flow and metabolism have been determined to be prion proteins (Table 1). A canonical example is the *S. cerevisiae* prion [*PSI*^*+*^], formed by the translation termination factor Sup35 [48]. At a low frequency, Sup35 converts from a soluble, functional conformation to a self-templating prion. This allows ribosomes to read through stop codons, uncovering previously silent genetic variation on a genome-wide level, and thus producing diverse and heritable phenotypes that are often disadvantageous but that can provide advantages in particular environments [158, 159]. This has led to the provocative hypothesis that yeast prions may serve as adaptive ‘bet-hedging’ elements to promote cellular survival in stressful environments [160]. In support of this was the discovery that hundreds of wild yeast strains contain heritable prion states, which frequently confer beneficial phenotypes under selective conditions [88].

Other notable examples of yeast prions that enable epigenetic switching to endow cells with beneficial phenotypes in specific metabolic and environmental conditions include [*URE3*] [47], [*MOT3*^+^] [88], and [*GAR*^+^] [161]. In particular, the [*SWI*^+^] prion formed by the yeast Swi1 protein represents an intriguing potential example of bet-hedging. As the main subunit of the SWI/SNF chromatin remodeling complex, Swi1 serves as a global transcriptional regulator. When Swi1 adopts its prion form, a variety of phenotypes can be induced, such as growth phenotypes on alternative carbon sources, sensitivity to antifungal agents, and, importantly, abolished adhesion to other cells or substrates [162]. By maintaining a small population of [*SWI*^+^] cells, an isogenic population can effectively safeguard against unpredictable environments via these diverse and potentially beneficial phenotypes, for example, by providing an opportunity for non-adherent [*SWI*^+^] cells to disperse to new locations to ensure re-population and survival [160].

Recently, the first bacterial protein capable of prion formation (the *Clostridium botulinum* global transcriptional terminator Rho [59]) and the first viral protein exhibiting prion-like self-propagating activity (formed by the baculovirus LEF-10 protein [78]) were discovered, suggesting that prion-based mechanisms for phenotypic diversification may be more pervasive than originally thought. We expect that more of these elements will be discovered with the development of new genetic tools to characterize and validate putative prions from other organisms [117, 163].

## Synthetic biology of protein assembly

**Fig. 3:**
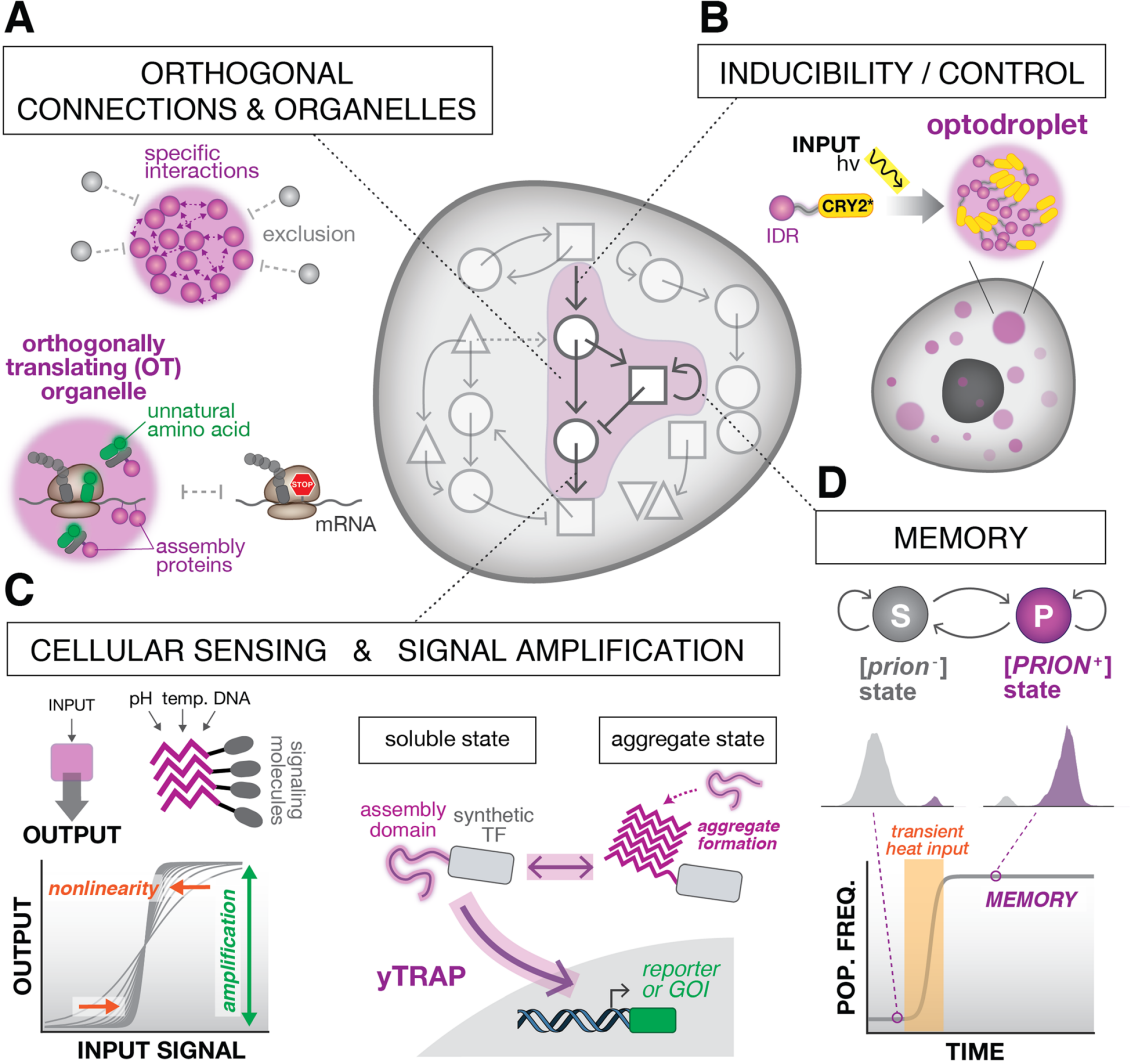
Application of higher-order protein assembly in synthetic biology. **a** Synthetic membraneless organelles, formed using proteins that undergo phase separation, can be used to enforce orthogonality of regulatory connections and biochemical reactions. This principle was recently used to create synthetic orthogonally translating (*OT*) organelles as sites for producing proteins that incorporate unnatural amino acids. **b** Exacting control over the formation of intracellular protein assemblies using optoDroplets. In this scheme, IDRs fused to light-inducible oligomerization domains enable the induction of phase separation by illumination with light. **c** Protein assembly systems as the basis of sensing and signal processing devices. *Left*: Protein assemblies can undergo dramatic changes in structure in response to small variations in environmental conditions, enabling exquisite sensing capabilities. *Right*: Changes in aggregation can be used to control downstream cellular processes. In the yTRAP system, the solubility state of an assembly domain is coupled to the activity of a synthetic TF and consequent activation of GOIs. **d** Prion proteins can exist stably in distinct conformational states, offering the potential to create synthetic memory devices based on prion switching

Synthetic biology aims to synthesize complex biological function using basic molecular parts, a bottom-up approach that has been productively used to study design principles of cellular systems and purposefully manipulate them for useful applications [164–168]. While the field has undergone tremendous growth [169], there are still many challenges and limitations for engineering biological systems with predictive and ambitious functions [170]. Relatedly, considerable biology remains largely untapped from an engineering perspective. One primary example is protein self-assembly and aggregation. Nature has ingeniously exploited this seemingly simple and ancient form of establishing molecular interactions to create emergent systems that accomplish many complex cellular tasks. Synthetic biologists would be well-served to bring these powerful elements into the engineering toolbox and to develop methods for manipulating protein assembly systems for study and application (Fig. 3).

### From sequence to behavior

One vision of synthetic biology is programming cellular behavior entirely at the level of DNA sequence. This presents an immediate challenge for engineering protein self-assembly systems, as these require an understanding of protein structure and stability and often rely on disordered and highly flexible structures. Indeed, while powerful software suites like Rosetta, I-TASSER, and QUARK can predict 3D structure of folded proteins based exclusively on amino acid sequence [171, 172], disordered proteins remain a challenge. To address this, computational approaches using hidden Markov models [173] and machine learning [174] have been developed to score propensity for properties, such as disorder [175], secondary structure [174], aggregation [176], prion behavior [68, 84–86, 177], and propensity to phase-separate [178, 179]. Genome-wide searches for PrDs utilizing such computational approaches have enabled the successful identification of new yeast prions, such as [*MOT*^*+*^] [68] and [*RNQ*^+^] [180].

One approach often used by synthetic biologists to engineer new synthetic protein systems is to leverage the functional modularity of certain protein domains. Fortunately, many PrDs have been found to be modular, which allows transfer of prion-forming capability onto new proteins by simply fusing them to PrDs [181]. These fusions can allow the activity of a protein to be regulated in a manner that depends on the prion conformation [68]. Moreover, because of the modular nature of PrDs, synthetic proteins harboring multiple PrDs could be generated, enabling artificial cross-seeding of aggregates and the study of higher-order prion interactions [117].

Another approach for engineering self-assembling protein systems based on sequence is to take advantage of studies using de novo peptides [182–184]. These studies have shown that tunable and reversible phase behaviors can, to some degree, be encoded into de novo synthetic proteins by combining low complexity domains in particular arrangements [183]. Using this knowledge, researchers have generated synthetic peptides with predictable phase behaviors [8, 182, 183, 185], synthetic prion domains [186], as well as redesigned bacterial amyloid proteins for application as structural biomaterials [187, 188], adhesives [189], and nanowires [190]. For example, researchers fused the *Escherichia coli* CsgA protein, responsible for curli amyloid fiber formation, to the mussel foot protein (Mfps) in order to create a synthetic protein capable of generating a fibrillary structure of adhesive proteins that outperforms mussel foot in adhesion to underwater surfaces [189].

### Making circuit and pathway connections

One focus of synthetic biology is the construction of genetic circuits, networks of interacting regulatory molecules that can manipulate information flow in living cells. Fundamental to genetic circuit design is the ability to make user-defined molecular interactions. Owing to the relative ease of engineering specific and orthogonal protein–DNA interactions, synthetic biologists have made great strides in building and exploring applications of transcriptional circuits [170, 191–197]. Building blocks for these circuits include naturally occurring microbial transcription factors (TFs) with well-defined DNA binding motifs as well as synthetic TFs that use programmable DNA-binding domains, such as zinc fingers, TALEs, or CRISPR/dCas9 [198–200]. Similarly, synthetic RNA-based circuits have been designed to great effect by exploiting simple Watson–Crick base-pairing interactions [201–204]. On the other hand, engineering protein-based systems, such as synthetic signaling circuits, has been more limited in part because of the inherent difficulty in making programmable protein–protein interactions [205, 206]. To date, synthetic biologists have relied on a relatively limited set of well-characterized, folded interaction domains to accomplish this task, for example, using leucine zippers and PDZ domains to enable recruitment of signaling proteins and pathway modulators [207–209]. More recent work has begun to expand this protein toolkit to viral proteases [210, 211] and even synthetic phospho-regulon motifs for building phosphorylation circuits [212].

Protein self-assembly could be used to enhance many of these efforts. For example, in eukaryotic transcriptional circuits, it is well-appreciated that genes are naturally regulated by large, multivalent TF complexes, rather than through the one-to-one interactions commonly used in synthetic circuits. The regulation of genes via multivalent assemblies provides opportunities to integrate many signals at a promoter and to achieve highly cooperative (‘digital’) transcriptional responses. In an exciting and extreme case of this, condensate formation at genomic loci has recently been shown to be associated with eukaryotic transcription initiation, and has been implicated in enabling highly cooperative and robust gene regulation [126, 137, 139]. Our group recently pioneered a method for engineering synthetic, multivalent TF complexes that utilize cooperative assembly to regulate genes, and showed that cooperative TF assemblies enable the construction of genetic circuits with complex signal processing behavior in yeast [192]. This framework could be extended to incorporate post-translationally regulated IDRs [213] in order to explore whether transcriptional condensates can be synthetically engineered and used to enable new forms of transcriptional control [214].

Protein self-assembly also represents an alternative mode of programming regulatory connections for synthetic protein circuits, one that can efficiently facilitate the creation of many multivalent interactions. For example, by creating fusions to modular IDRs, signaling proteins can be directed to scaffold or phase separate, thereby increasing the specificity and efficiency of a signaling task. Moreover, the formation of these assemblies can be controlled in a variety of ways. One interesting way is through post-translational modifications, which are known to dramatically alter the biophysical properties of an IDR. For example, phosphorylation can dismantle RNA granules [215], while methylation [216], acetylation [213], and SUMOylation [217] can promote the dissolution of several types of condensates. Exploiting these post-translational modifications, either via synthetic or endogenous mechanisms, could provide a means to control the formation and reversal of protein assemblies that bring together regulatory proteins of interest.

### Constructing synthetic organelles

The ability to control the formation of assemblies offers the intriguing possibility for compartmentalizing biochemical reactions into spatially separated “synthetic organelles”. These could be programmed to serve as factories for the production of complex chemicals [218] or signal transduction hubs in synthetic signaling circuits, or spatially separate synthetic processing units inside the cell. This strategy offers a number of unique advantages for synthetic biology. First, constraining reaction components into a small compartment creates a “reaction crucible” that can increase the efficiency of enzymatic reactions [219]. Second, biochemical specificity is inherently enforced by spatially separated compartments, thus effectively insulating synthetic circuits and addressing a key challenge in synthetic biology of component cross-talk. Finally, multiple co-existing assemblies could in principle be encoded in a single cell, each performing new and different reactions. In a very recent and striking example of this concept, researchers designed an artificial membraneless organelle capable of sequestering and supporting orthogonal protein translation machinery (mRNA, suppressor tRNA, unnatural amino acid tRNA synthetase, and ribosomes) to efficiently produce proteins that incorporate unnatural amino acids [220] (Fig. 3).

The formation of intracellular organelles can be synthetically and spatiotemporally controlled by new methods, such as optoDroplets [219]. OptoDroplets are composed of a protein fusion between the IDRs of RBPs, such as FUS and DDX4, and the blue-light inducible oligomerization domain CRY2. Light-stimulated oligomerization of CRY2 serves to increase local concentration and nucleate the formation of assemblies. Critically, the assembly properties of optoDroplets can be adjusted based on the protein fusion and the light stimulation. Low intensity light and short exposures lead to reversible droplets, whereas high intensity or increased exposure induces formation of more stable, amyloid-like aggregates.

Synthetic organelles that carry out desired reactions can also be engineered with other classes of self-assembling proteins, including modular folded proteins. One notable example involves the encapsulin family of proteins [221]. These bacterial proteins assemble into large, hollow nanocompartments, which can be loaded with cargo proteins that have been equipped with an encapsulation tag [222]. By tagging enzymes of a biochemical pathway, a desired reaction can be physically constrained and efficiently performed within the nanocompartment [223].

### Cellular sensing and signal processing

Cells have exquisite sensitivity for a diverse array of chemical and biological stimuli and the ability to actuate appropriate responses to these signals. Synthetic biologists aim to co-opt these systems in order to create engineered cellular sensors and signal processing devices that are responsive to desired ligands and stimuli. Protein assembly systems have unique features that can potentiate these efforts. For example, protein assemblies can undergo dramatic changes in structure in response to small variations in environmental conditions. A striking example of this exquisite sensitivity involves the yeast stress granule polyA-binding protein 1 (Pab1), which undergoes phase separation and hydrogel formation in response to increases in temperature [23]. Specifically, in cells, this protein is soluble in the cytoplasm at 30 °C and readily forms droplets at 46 °C. Within this physiological range of temperatures, Pab1 was shown to form droplets of increasing size as temperature was increased in in vitro studies.

In addition to considering assemblies as direct sensors of physiological changes, significant technological efforts are underway to enable sensitive detection and processing of aggregation states inside living cells. One recently developed technology, termed distributed amphifluoric FRET (DamFRET), uses fusions to a photoconvertible fluorophore to quantify protein aggregation states based on FRET signal [32]. DamFRET can provide information about the proximity and conformation of protein monomers as a function of their concentration. As such, it is useful for quantifying the kinetics of nucleation of a protein aggregate in cells. Nucleation is a rare, kinetically slow, and unfavorable step that precedes a highly favorable elongation step, leading to a stable aggregate. Since most proteins exist in solution at concentrations near the nucleation barrier, stochastic nucleation events may occur. By increasing protein concentration, the probability of nucleation events increases in a way that is intimately related to the sequence properties of the biomolecule. By evaluating how mutations in protein sequence affect the critical concentration for nucleation, DamFRET experiments are able to elucidate the sequence properties that affect nucleation kinetics and therefore favor or disfavor protein aggregation.

Changes in aggregation can also be coupled to and used to control downstream cellular processes. One example of this capability was demonstrated in the design of the yTRAP (yeast Transcriptional Reporting of Aggregating Protein) system, a genetic tool we developed for high-throughput sensing and control of protein aggregation states in yeast cells [117] (Fig. 3). Specifically, by fusing an aggregation-prone protein domain of interest to a synthetic TF, the activity of the synthetic TF is coupled to the solubility state of the protein domain of interest. Therefore, in the “soluble state”, the yTRAP module is free to regulate its cognate synthetic reporter locus, whereas in the “aggregated state”, the module participates in cellular aggregation quantitatively affecting the transcriptional activation. This framework enables high-throughput genetic and chemical screens to discover aggregation-prone domains and modulators of their aggregation. Additionally, this mechanism could be adapted to create more elaborate synthetic protein assembly systems that control other cellular processes.

### Engineering memory and inheritance

Memory is fundamental to computation by man-made devices. Similarly, the ability to store memory of past events is a universal feature of living systems, and is a requirement for a number of fundamental biological processes, such as environmental adaptation, cellular differentiation, and multicellular development. Biological memory in this case is defined as the conversion of a transient signal into a sustained response. Implementing synthetic systems that achieve cellular memory has been a long-standing goal of synthetic biologists, dating back to the origins of the field. Among the first artificial genetic circuits reported was the genetic toggle switch, in which two bacterial transcriptional repressors were arranged in a mutual inhibitory network to give rise to bistability, i.e., a system that can switch between two stable states [224]. Since then, many other molecular mechanisms for encoding cellular memory, naturally inspired or otherwise, have been implemented and explored [225, 226]. These can be broadly divided between epigenetic (transcriptional feedback loops, heritable chromatin changes, etc.) [227–229] and inducible DNA mutations/alterations [230–234]. Taken together, this work has yielded foundational synthetic elements for building more sophisticated biological systems that enable cell state changes, memory of gene expression states, and cellular devices that record lineage and environmental information.

Yeast prions have several properties that, in principle, make them excellent candidates for building stable synthetic memory [225]. First, they exhibit bistability, meaning that a given cell can stably exist in either a [*prion*^*−*^] or [*PRION*^*+*^] phenotypic state. Second, cells can reversibly transition between the two stable states in all-or-none fashion [235]. Third, the states propagate for long biological time scales: because the aggregated prion conformation is transmitted through the cytoplasm, these confomers and their associated phenotypes are robustly inherited by progeny. Taken together, this forms the basis of bistable switches that can set, reset, and store long-lived biological memory [236]. These capabilities were recently demonstrated in the construction of a synthetic memory device that recorded a transient environmental event into a population of yeast cells using prion switching [117]. Specifically, by placing expression of a novel [*PSI*^+^]-inducing factor under the control of a temperature-sensitive promoter, cells could be programmed to remember a short exposure to elevated temperature over ten generations later. These types of prion-based memory elements could be deployed in populations to record and report on environmental variables experienced in natural or industrial contexts, such as in industrial bioreactors.

Prion conformations and their associated phenotypes are inherited in a dominant non-Mendelian fashion [48]. As such, they effectively act as the epigenetic analogs of gene drives, genetic systems that bias the standard Mendelian inheritance of a specific allele to increase its prevalence in a population [237]. Manipulating these elements could thus open up the possibility for driving or reshaping the inheritance of an epigenetic trait in a population. As a first step toward this goal, prion alleles with the propensity to cure prions were identified and used to construct anti-prion drives (APDs), systems that can reverse the dominant inheritance of prions (and in some cases eliminate them) [117]. In the future, engineered strains carrying prion and APD elements could be deployed in wild-type populations to compete and perform population-level control of desired epigenetic traits. Building out this toolkit of manipulable prion-like elements should provide synthetic biologists with new strategies for engineering computational and evolutionary functions into cells and populations.

## Concluding remarks

Protein self-assembly remains a little explored and exploited mechanism for synthetic biology and cellular engineering; but it could offer many advantages, such as the ability to program emergent nonlinear behaviors and catalyze drastic cellular changes with a relatively small set of constituent parts. However, the unique properties of these systems can also make them difficult to design and manipulate. For example, challenges associated with designing and manipulating disordered and self-assembling proteins include: (1) The high false positive rate of structure prediction algorithms. This necessitates experimental validation of assembly formation for each predicted protein or domain. (2) Predicting and designing the stoichiometry of disordered protein assemblies. This will require an increased understanding of the basic biophysics of assembly formation, or either focusing on applications that are insensitive to stoichiometry or using structured protein domains to introduce well-defined molecular interactions. (3) Lack of methods for precisely controlling formation and dissociation of assemblies. Here, the use of engineered post-translation modifications known to modulate assembly formation could be highly advantageous, but will require the development of synthetic tools for exacting control over these signaling events. Overall, through the combination of synthetic biology manipulation, quantitative studies, and an increased understanding of their underlying biophysics, we can make possible an era of creating designer protein assemblies for application and study.

## References

[1] Hyman AA, Weber CA, Julicher F (2014). Liquid-liquid phase separation in biology. Annu Rev Cell Dev Biol.

[2] Harvey ZH, Chen Y, Jarosz DF (2018). Protein-based inheritance: epigenetics beyond the chromosome. Mol Cell.

[3] Jakobson CM, Jarosz DF (2018). Organizing biochemistry in space and time using prion-like self-assembly. Curr Opin Systems Biol.

[4] Mitrea DM, Kriwacki RW (2016). Phase separation in biology; functional organization of a higher order. Cell Commun Signaling.

[5] Wilson CJ, Bommarius AS, Champion JA, Chernoff YO, Lynn DG, Paravastu AK, Liang C, Hsieh MC, Heemstra JM (2018). Biomolecular assemblies: moving from observation to predictive design. Chem Rev.

[6] Tompa P, Schad E, Tantos A, Kalmar L (2015). Intrinsically disordered proteins: emerging interaction specialists. Curr Opin Struct Biol.

[7] Banjade S, Wu Q, Mittal A, Peeples WB, Pappu RV, Rosen MK (2015). Conserved interdomain linker promotes phase separation of the multivalent adaptor protein Nck. Proc Natl Acad Sci U S A.

[8] Li P, Banjade S, Cheng HC, Kim S, Chen B, Guo L, Llaguno M, Hollingsworth JV, King DS, Banani SF (2012). Phase transitions in the assembly of multivalent signalling proteins. Nature..

[9] Banjade S, Rosen MK. Phase transitions of multivalent proteins can promote clustering of membrane receptors. eLife. 2014;3:e04123. 10.7554/eLife.04123. 10.7554/eLife.04123 PMC4238058 25321392

[10] Molliex A, Temirov J, Lee J, Coughlin M, Kanagaraj AP, Kim HJ, Mittag T, Taylor JP (2015). Phase separation by low complexity domains promotes stress granule assembly and drives pathological fibrillization. Cell..

[11] Martin EW, Mittag T (2018). Relationship of sequence and phase separation in protein low-complexity regions. Biochemistry..

[12] Brangwynne CP, Tompa P, Pappu RV (2015). Polymer physics of intracellular phase transitions. Nat Phys.

[13] Lin YH, Forman-Kay JD, Chan HS (2018). Theories for sequence-dependent phase behaviors of biomolecular condensates. Biochemistry..

[14] Boeynaems S, Alberti S, Fawzi NL, Mittag T, Polymenidou M, Rousseau F, Schymkowitz J, Shorter J, Wolozin B, Van Den Bosch L (2018). Protein phase separation: a new phase in cell biology. Trends Cell Biol.

[15] Holehouse AS, Pappu RV (2018). Functional implications of intracellular phase transitions. Biochemistry..

[16] Alberti S, Gladfelter A, Mittag T (2019). Considerations and challenges in studying liquid-liquid phase separation and biomolecular condensates. Cell..

[17] Banani SF, Lee HO, Hyman AA, Rosen MK (2017). Biomolecular condensates: organizers of cellular biochemistry. Nat Rev Mol Cell Biol..

[18] Brangwynne CP, Mitchison TJ, Hyman AA (2011). Active liquid-like behavior of nucleoli determines their size and shape in Xenopus laevis oocytes. Proc Natl Acad Sci U S A.

[19] Brangwynne CP, Eckmann CR, Courson DS, Rybarska A, Hoege C, Gharakhani J, Julicher F, Hyman AA (2009). Germline P granules are liquid droplets that localize by controlled dissolution/condensation. Science..

[20] Kroschwald S, Maharana S, Mateju D, Malinovska L, Nuske E, Poser I, Richter D, Alberti S (2015). Promiscuous interactions and protein disaggregases determine the material state of stress-inducible RNP granules. eLife..

[21] Zhu L, Brangwynne CP (2015). Nuclear bodies: the emerging biophysics of nucleoplasmic phases. Curr Opin Cell Biol.

[22] Feric M, Vaidya N, Harmon TS, Mitrea DM, Zhu L, Richardson TM, Kriwacki RW, Pappu RV, Brangwynne CP (2016). Coexisting liquid phases underlie nucleolar subcompartments. Cell..

[23] Riback JA, Katanski CD, Kear-Scott JL, Pilipenko EV, Rojek AE, Sosnick TR, Drummond DA (2017). Stress-triggered phase separation is an adaptive, evolutionarily tuned response. Cell..

[24] Cid-Samper F, Gelabert-Baldrich M, Lang B, Lorenzo-Gotor N, Ponti RD, Severijnen LAWFM, et al. An Integrative Study of Protein-RNA Condensates Identifies Scaffolding RNAs and Reveals Players in Fragile X-Associated Tremor/Ataxia Syndrome. Cell Rep. 2018;25:3422-3434.e7. 10.1016/j.celrep.2018.11.076. 10.1016/j.celrep.2018.11.076 PMC6315285 30566867

[25] Pak CW, Kosno M, Holehouse AS, Padrick SB, Mittal A, Ali R, Yunus AA, Liu DR, Pappu RV, Rosen MK (2016). Sequence determinants of intracellular phase separation by complex coacervation of a disordered protein. Mol Cell.

[26] Lin YH, Forman-Kay JD, Chan HS (2016). Sequence-specific polyampholyte phase separation in membraneless organelles. Phys Rev Lett.

[27] Wang J, Choi JM, Holehouse AS, Lee HO, Zhang X, Jahnel M, Maharana S, Lemaitre R, Pozniakovsky A, Drechsel D (2018). A molecular grammar governing the driving forces for phase separation of prion-like RNA binding proteins. Cell..

[28] Baldwin AJ, Knowles TP, Tartaglia GG, Fitzpatrick AW, Devlin GL, Shammas SL, Waudby CA, Mossuto MF, Meehan S, Gras SL (2011). Metastability of native proteins and the phenomenon of amyloid formation. J Am Chem Soc.

[29] Greenwald J, Riek R (2010). Biology of amyloid: structure, function, and regulation. Structure..

[30] Landrum E, Wetzel R (2014). Biophysical underpinnings of the repeat length dependence of polyglutamine amyloid formation. J Biol Chem.

[31] Knowles TP, Waudby CA, Devlin GL, Cohen SI, Aguzzi A, Vendruscolo M, Terentjev EM, Welland ME, Dobson CM (2009). An analytical solution to the kinetics of breakable filament assembly. Science..

[32] Khan T, Kandola TS, Wu J, Venkatesan S, Ketter E, Lange JJ, Rodriguez Gama A, Box A, Unruh JR, Cook M (2018). Quantifying nucleation in vivo reveals the physical basis of prion-like phase behavior. Mol Cell.

[33] Narayanan A, Meriin A, Andrews JO, Spille JH, Sherman MY, Cisse II. A first order phase transition mechanism underlies protein aggregation in mammalian cells. eLife. 2019;8:e39695. 10.7554/eLife.39695. 10.7554/eLife.39695 PMC6361590 30716021

[34] Maurer-Stroh S, Debulpaep M, Kuemmerer N, Lopez de la Paz M, Martins IC, Reumers J, Morris KL, Copland A, Serpell L, Serrano L et al. Exploring the sequence determinants of amyloid structure using position-specific scoring matrices. Nat Methods 2010;7(3):237–242. 10.1038/nmeth.1432 20154676

[35] Goldschmidt L, Teng PK, Riek R, Eisenberg D (2010). Identifying the amylome, proteins capable of forming amyloid-like fibrils. Proc Natl Acad Sci U S A.

[36] Knowles TP, Buehler MJ (2011). Nanomechanics of functional and pathological amyloid materials. Nat Nanotechnol.

[37] Tanaka M, Collins SR, Toyama BH, Weissman JS (2006). The physical basis of how prion conformations determine strain phenotypes. Nature..

[38] Petkova AT, Leapman RD, Guo Z, Yau WM, Mattson MP, Tycko R (2005). Self-propagating, molecular-level polymorphism in Alzheimer's beta-amyloid fibrils. Science..

[39] Mukherjee A, Morales-Scheihing D, Butler PC, Soto C (2015). Type 2 diabetes as a protein misfolding disease. Trends Mol Med.

[40] Lachmann HJ, Hawkins PN (2006). Systemic amyloidosis. Curr Opin Pharmacol.

[41] Buxbaum JN, Linke RP (2012). A molecular history of the amyloidoses. J Mol Biol.

[42] Jarosz DF, Khurana V (2017). Specification of physiologic and disease states by distinct proteins and protein conformations. Cell..

[43] Scheckel C, Aguzzi A (2018). Prions, prionoids and protein misfolding disorders. Nat Rev Genet..

[44] Knowles TP, Vendruscolo M, Dobson CM (2014). The amyloid state and its association with protein misfolding diseases. Nat Rev Mol Cell Biol.

[45] Prusiner SB (1982). Novel proteinaceous infectious particles cause scrapie. Science..

[46] Prusiner SB (1986). Prions are novel infectious pathogens causing scrapie and Creutzfeldt-Jakob disease. BioEssays..

[47] Shorter J, Lindquist S (2005). Prions as adaptive conduits of memory and inheritance. Nat Rev Genet.

[48] Wickner RB (1994). [URE3] as an altered URE2 protein: evidence for a prion analog in Saccharomyces cerevisiae. Science..

[49] Holmes BB, Diamond MI (2014). Prion-like properties of tau protein: the importance of extracellular tau as a therapeutic target. J Biol Chem.

[50] Jackson GS, Hosszu LL, Power A, Hill AF, Kenney J, Saibil H, Craven CJ, Waltho JP, Clarke AR, Collinge J (1999). Reversible conversion of monomeric human prion protein between native and fibrilogenic conformations. Science..

[51] Kaganovich D, Kopito R, Frydman J (2008). Misfolded proteins partition between two distinct quality control compartments. Nature..

[52] Chen B, Retzlaff M, Roos T, Frydman J (2011). Cellular strategies of protein quality control. Cold Spring Harb Perspect Biol.

[53] Franzmann TM, Jahnel M, Pozniakovsky A, Mahamid J, Holehouse AS, Nuske E, Richter D, Baumeister W, Grill SW, Pappu RV (2018). Phase separation of a yeast prion protein promotes cellular fitness. Science.

[54] Brown JC, Lindquist S (2009). A heritable switch in carbon source utilization driven by an unusual yeast prion. Genes Dev.

[55] Roberts BT, Wickner RB (2003). Heritable activity: a prion that propagates by covalent autoactivation. Genes Dev.

[56] Majumdar A, Cesario WC, White-Grindley E, Jiang H, Ren F, Khan MR, Li L, Choi EM, Kannan K, Guo F (2012). Critical role of amyloid-like oligomers of Drosophila Orb2 in the persistence of memory. Cell..

[57] Stephan JS, Fioriti L, Lamba N, Colnaghi L, Karl K, Derkatch IL, Kandel ER (2015). The CPEB3 protein is a functional prion that interacts with the actin cytoskeleton. Cell Rep.

[58] Si K, Kandel ER (2016). The role of functional prion-like proteins in the persistence of memory. Cold Spring Harb Perspect Biol.

[59] Yuan AH, Hochschild A (2017). A bacterial global regulator forms a prion. Science..

[60] Crow ET, Li L (2011). Newly identified prions in budding yeast, and their possible functions. Semin Cell Dev Biol.

[61] Wickner RB (1997). A new prion controls fungal cell fusion incompatibility. Proc Natl Acad Sci U S A.

[62] Saupe SJ (2011). The [Het-s] prion of Podospora anserina and its role in heterokaryon incompatibility. Semin Cell Dev Biol.

[63] Antonets KS, Nizhnikov AA (2017). Amyloids and prions in plants: facts and perspectives. Prion..

[64] Chakrabortee S, Kayatekin C, Newby GA, Mendillo ML, Lancaster A, Lindquist S (2016). Luminidependens (LD) is an Arabidopsis protein with prion behavior. Proc Natl Acad Sci U S A.

[65] Chernova TA, Kiktev DA, Romanyuk AV, Shanks JR, Laur O, Ali M, Ghosh A, Kim D, Yang Z, Mang M (2017). Yeast short-lived actin-associated protein forms a metastable prion in response to thermal stress. Cell Rep.

[66] Suzuki G, Shimazu N, Tanaka M (2012). A yeast prion, Mod5, promotes acquired drug resistance and cell survival under environmental stress. Science..

[67] Wickner RB, Shewmaker FP, Bateman DA, Edskes HK, Gorkovskiy A, Dayani Y, Bezsonov EE (2015). Yeast prions: structure, biology, and prion-handling systems. Microbiol Mol Biol Rev.

[68] Alberti S, Halfmann R, King O, Kapila A, Lindquist S (2009). A systematic survey identifies prions and illuminates sequence features of prionogenic proteins. Cell..

[69] Saifitdinova AF, Nizhnikov AA, Lada AG, Rubel AA, Magomedova ZM, Ignatova VV, Inge-Vechtomov SG, Galkin AP (2010). [NSI (+)]: a novel non-Mendelian nonsense suppressor determinant in Saccharomyces cerevisiae. Curr Genet.

[70] Harbi D, Harrison PM (2014). Interaction networks of prion, prionogenic and prion-like proteins in budding yeast, and their role in gene regulation. PLoS One.

[71] Halfmann R, Wright JR, Alberti S, Lindquist S, Rexach M (2012). Prion formation by a yeast GLFG nucleoporin. Prion..

[72] Patel BK, Gavin-Smyth J, Liebman SW (2009). The yeast global transcriptional co-repressor protein Cyc8 can propagate as a prion. Nat Cell Biol.

[73] Liebman SW, Chernoff YO (2012). Prions in yeast. Genetics..

[74] MacLea KS, Ross ED (2011). Strategies for identifying new prions in yeast. Prion..

[75] Wulf MA, Senatore A, Aguzzi A (2017). The biological function of the cellular prion protein: an update. BMC Biol.

[76] Cai X, Chen J, Xu H, Liu S, Jiang QX, Halfmann R, Chen ZJ (2014). Prion-like polymerization underlies signal transduction in antiviral immune defense and inflammasome activation. Cell..

[77] Franklin BS, Bossaller L, De Nardo D, Ratter JM, Stutz A, Engels G, Brenker C, Nordhoff M, Mirandola SR, Al-Amoudi A (2014). The adaptor ASC has extracellular and 'prionoid' activities that propagate inflammation. Nat Immunol.

[78] Nan H, Chen H, Tuite MF, Xu X (2019). A viral expression factor behaves as a prion. Nat Commun.

[79] Hou F, Sun L, Zheng H, Skaug B, Jiang QX, Chen ZJ (2011). MAVS forms functional prion-like aggregates to activate and propagate antiviral innate immune response. Cell..

[80] Inoue Y, Kawai-Noma S, Koike-Takeshita A, Taguchi H, Yoshida M (2011). Yeast prion protein New1 can break Sup35 amyloid fibrils into fragments in an ATP-dependent manner. Genes Cells.

[81] Chakrabortee S, Byers JS, Jones S, Garcia DM, Bhullar B, Chang A, She R, Lee L, Fremin B, Lindquist S (2016). Intrinsically disordered proteins drive emergence and inheritance of biological traits. Cell..

[82] Wickner RB (2016). Yeast and fungal prions. Cold Spring Harb Perspect Biol.

[83] Michelitsch MD, Weissman JS (2000). A census of glutamine/asparagine-rich regions: implications for their conserved function and the prediction of novel prions. Proc Natl Acad Sci U S A.

[84] Espinosa Angarica V, Ventura S, Sancho J (2013). Discovering putative prion sequences in complete proteomes using probabilistic representations of Q/N-rich domains. BMC Genomics.

[85] Cascarina SM, Ross ED (2014). Yeast prions and human prion-like proteins: sequence features and prediction methods. Cell Mol Life Sci.

[86] Lancaster AK, Nutter-Upham A, Lindquist S, King OD (2014). PLAAC: a web and command-line application to identify proteins with prion-like amino acid composition. Bioinformatics..

[87] Chernoff YO, Lindquist SL, Ono B, Inge-Vechtomov SG, Liebman SW (1995). Role of the chaperone protein Hsp104 in propagation of the yeast prion-like factor [psi+]. Science..

[88] Halfmann R, Jarosz DF, Jones SK, Chang A, Lancaster AK, Lindquist S (2012). Prions are a common mechanism for phenotypic inheritance in wild yeasts. Nature..

[89] Diaz-Avalos R, King CY, Wall J, Simon M, Caspar DL (2005). Strain-specific morphologies of yeast prion amyloid fibrils. Proc Natl Acad Sci U S A.

[90] Toyama BH, Kelly MJ, Gross JD, Weissman JS (2007). The structural basis of yeast prion strain variants. Nature..

[91] Uptain SM, Sawicki GJ, Caughey B, Lindquist S (2001). Strains of [PSI(+)] are distinguished by their efficiencies of prion-mediated conformational conversion. EMBO J.

[92] Aguzzi A (2008). Unraveling prion strains with cell biology and organic chemistry. Proc Natl Acad Sci U S A.

[93] Scott M, Foster D, Mirenda C, Serban D, Coufal F, Walchli M, Torchia M, Groth D, Carlson G, DeArmond SJ (1989). Transgenic mice expressing hamster prion protein produce species-specific scrapie infectivity and amyloid plaques. Cell..

[94] Tanaka M, Chien P, Naber N, Cooke R, Weissman JS (2004). Conformational variations in an infectious protein determine prion strain differences. Nature..

[95] Ghosh R, Dong J, Wall J, Frederick KK. Amyloid fibrils embodying distinctive yeast prion phenotypes exhibit diverse morphologies. FEMS Yeast Res. 2018;18(6). doi: 10.1093/femsyr/foy059. 10.1093/femsyr/foy059 PMC6001884 29846554

[96] Bateman DA, Wickner RB (2013). The [PSI+] prion exists as a dynamic cloud of variants. PLoS Genet.

[97] Frederick KK, Debelouchina GT, Kayatekin C, Dorminy T, Jacavone AC, Griffin RG, Lindquist S (2014). Distinct prion strains are defined by amyloid core structure and chaperone binding site dynamics. Chem Biol.

[98] Soto C, Pritzkow S (2018). Protein misfolding, aggregation, and conformational strains in neurodegenerative diseases. Nat Neurosci.

[99] Lopez-Otin C, Blasco MA, Partridge L, Serrano M, Kroemer G (2013). The hallmarks of aging. Cell..

[100] Groenning M (2010). Binding mode of Thioflavin T and other molecular probes in the context of amyloid fibrils-current status. J Chem Biol.

[101] Buell AK, Galvagnion C, Gaspar R, Sparr E, Vendruscolo M, Knowles TP, Linse S, Dobson CM (2014). Solution conditions determine the relative importance of nucleation and growth processes in alpha-synuclein aggregation. Proc Natl Acad Sci U S A.

[102] Navarro S, Ventura S (2014). Fluorescent dye ProteoStat to detect and discriminate intracellular amyloid-like aggregates in Escherichia coli. Biotechnol J.

[103] Halfmann R, Lindquist S (2008). Screening for amyloid aggregation by semi-denaturing detergent-agarose gel electrophoresis. J Vis Exp.

[104] Sunde M, Serpell LC, Bartlam M, Fraser PE, Pepys MB, Blake CC (1997). Common core structure of amyloid fibrils by synchrotron X-ray diffraction. J Mol Biol.

[105] Tycko R (2011). Solid-state NMR studies of amyloid fibril structure. Annu Rev Phys Chem.

[106] Lu JX, Qiang W, Yau WM, Schwieters CD, Meredith SC, Tycko R (2013). Molecular structure of beta-amyloid fibrils in Alzheimer's disease brain tissue. Cell..

[107] Frederick KK, Michaelis VK, Corzilius B, Ong TC, Jacavone AC, Griffin RG, Lindquist S (2015). Sensitivity-enhanced NMR reveals alterations in protein structure by cellular milieus. Cell..

[108] Fawzi NL, Ying J, Torchia DA, Clore GM (2010). Kinetics of amyloid beta monomer-to-oligomer exchange by NMR relaxation. J Am Chem Soc.

[109] Eftekharzadeh B, Piai A, Chiesa G, Mungianu D, Garcia J, Pierattelli R, Felli IC, Salvatella X (2016). Sequence context influences the structure and aggregation behavior of a PolyQ tract. Biophys J.

[110] Theillet FX, Binolfi A, Bekei B, Martorana A, Rose HM, Stuiver M, Verzini S, Lorenz D, van Rossum M, Goldfarb D (2016). Structural disorder of monomeric alpha-synuclein persists in mammalian cells. Nature..

[111] Kaminski CF, Kaminski Schierle GS (2016). Probing amyloid protein aggregation with optical superresolution methods: from the test tube to models of disease. Neurophotonics..

[112] Gremer L, Scholzel D, Schenk C, Reinartz E, Labahn J, Ravelli RBG, Tusche M, Lopez-Iglesias C, Hoyer W, Heise H (2017). Fibril structure of amyloid-beta(1-42) by cryo-electron microscopy. Science..

[113] Guerrero-Ferreira R, Taylor NM, Mona D, Ringler P, Lauer ME, Riek R, Britschgi M, Stahlberg H. Cryo-EM structure of alpha-synuclein fibrils. eLife. 2018;7:e36402. 10.7554/eLife.36402. 10.7554/eLife.36402 PMC6092118 29969391

[114] Dine E, Toettcher JE (2018). Optogenetic reconstitution for determining the form and function of membraneless organelles. Biochemistry..

[115] Heilemann M, van de Linde S, Schuttpelz M, Kasper R, Seefeldt B, Mukherjee A, Tinnefeld P, Sauer M (2008). Subdiffraction-resolution fluorescence imaging with conventional fluorescent probes. Angew Chem.

[116] Kaminski Schierle GS, van de Linde S, Erdelyi M, Esbjorner EK, Klein T, Rees E, Bertoncini CW, Dobson CM, Sauer M, Kaminski CF (2011). In situ measurements of the formation and morphology of intracellular beta-amyloid fibrils by super-resolution fluorescence imaging. J Am Chem Soc.

[117] Newby GA, Kiriakov S, Hallacli E, Kayatekin C, Tsvetkov P, Mancuso CP, Bonner JM, Hesse WR, Chakrabortee S, Manogaran AL (2017). A genetic tool to track protein aggregates and control prion inheritance. Cell..

[118] Pereira M, Tome D, Domingues AS, Varanda AS, Paulo C, Santos MAS, Soares AR (2018). A fluorescence-based sensor assay that monitors general protein aggregation in human cells. Biotechnol J.

[119] Zeng Y, Jones AM, Thomas EE, Nassif B, Silberg JJ, Segatori L (2018). A split transcriptional repressor that links protein solubility to an orthogonal genetic circuit. ACS Synthetic Biol..

[120] Saunders JC, Young LM, Mahood RA, Jackson MP, Revill CH, Foster RJ, Smith DA, Ashcroft AE, Brockwell DJ, Radford SE (2016). An in vivo platform for identifying inhibitors of protein aggregation. Nat Chem Biol.

[121] Holmes DL, Lancaster AK, Lindquist S, Halfmann R (2013). Heritable remodeling of yeast multicellularity by an environmentally responsive prion. Cell..

[122] Du Z, Park KW, Yu H, Fan Q, Li L (2008). Newly identified prion linked to the chromatin-remodeling factor Swi1 in Saccharomyces cerevisiae. Nat Genet.

[123] Talarek N, Maillet L, Cullin C, Aigle M (2005). The [URE3] prion is not conserved among Saccharomyces species. Genetics..

[124] Satpute-Krishnan P, Serio TR (2005). Prion protein remodelling confers an immediate phenotypic switch. Nature..

[125] Hofmann J, Vorberg I (2013). Life cycle of cytosolic prions. Prion..

[126] Cho WK, Spille JH, Hecht M, Lee C, Li C, Grube V, Cisse II (2018). Mediator and RNA polymerase II clusters associate in transcription-dependent condensates. Science..

[127] Boija A, Klein IA, Sabari BR, Dall'Agnese A, Coffey EL, Zamudio AV, Li CH, Shrinivas K, Manteiga JC, Hannett NM (2018). Transcription factors activate genes through the phase-separation capacity of their activation domains. Cell..

[128] Li J, McQuade T, Siemer AB, Napetschnig J, Moriwaki K, Hsiao YS, Damko E, Moquin D, Walz T, McDermott A (2012). The RIP1/RIP3 necrosome forms a functional amyloid signaling complex required for programmed necrosis. Cell..

[129] Dick MS, Sborgi L, Ruhl S, Hiller S, Broz P (2016). ASC filament formation serves as a signal amplification mechanism for inflammasomes. Nat Commun.

[130] Caudron F, Barral Y (2013). A super-assembly of Whi3 encodes memory of deceptive encounters by single cells during yeast courtship. Cell..

[131] Sudhakaran IP, Ramaswami M (2017). Long-term memory consolidation: the role of RNA-binding proteins with prion-like domains. RNA Biol.

[132] Kim Y, Furman CM, Manhart CM, Alani E, Finkelstein IJ (2019). Intrinsically disordered regions regulate both catalytic and non-catalytic activities of the MutLalpha mismatch repair complex. Nucleic Acids Res.

[133] Carpenter K, Bell RB, Yunus J, Amon A, Berchowitz LE (2018). Phosphorylation-mediated clearance of amyloid-like assemblies in meiosis. Dev Cell.

[134] Guillen-Boixet J, Buzon V, Salvatella X, Mendez R. CPEB4 is regulated during cell cycle by ERK2/Cdk1-mediated phosphorylation and its assembly into liquid-like droplets. eLife. 2016;5:e19298. 10.7554/eLife.19298. 10.7554/eLife.19298 PMC5089860 27802129

[135] Berchowitz LE, Kabachinski G, Walker MR, Carlile TM, Gilbert WV, Schwartz TU, Amon A (2015). Regulated formation of an amyloid-like translational repressor governs gametogenesis. Cell..

[136] Halfmann R, Lindquist S (2010). Epigenetics in the extreme: prions and the inheritance of environmentally acquired traits. Science..

[137] Hnisz D, Shrinivas K, Young RA, Chakraborty AK, Sharp PA (2017). A phase separation model for transcriptional control. Cell..

[138] Sabari BR, Dall'Agnese A, Boija A, Klein IA, Coffey EL, Shrinivas K, Abraham BJ, Hannett NM, Zamudio AV, Manteiga JC (2018). Coactivator condensation at super-enhancers links phase separation and gene control. Science.

[139] Lu H, Yu D, Hansen AS, Ganguly S, Liu R, Heckert A, Darzacq X, Zhou Q (2018). Phase-separation mechanism for C-terminal hyperphosphorylation of RNA polymerase II. Nature..

[140] Larson AG, Elnatan D, Keenen MM, Trnka MJ, Johnston JB, Burlingame AL, Agard DA, Redding S, Narlikar GJ (2017). Liquid droplet formation by HP1alpha suggests a role for phase separation in heterochromatin. Nature..

[141] Strom AR, Emelyanov AV, Mir M, Fyodorov DV, Darzacq X, Karpen GH (2017). Phase separation drives heterochromatin domain formation. Nature..

[142] Canzio D, Liao M, Naber N, Pate E, Larson A, Wu S, Marina DB, Garcia JF, Madhani HD, Cooke R (2013). A conformational switch in HP1 releases auto-inhibition to drive heterochromatin assembly. Nature..

[143] Weber SC, Brangwynne CP (2012). Getting RNA and protein in phase. Cell..

[144] Sheu-Gruttadauria J, MacRae IJ (2018). Phase transitions in the assembly and function of human miRISC. Cell..

[145] Harrison AF, Shorter J (2017). RNA-binding proteins with prion-like domains in health and disease. Biochem J.

[146] Jain A, Vale RD (2017). RNA phase transitions in repeat expansion disorders. Nature..

[147] Wu H, Fuxreiter M (2016). The structure and dynamics of higher-order assemblies: amyloids, signalosomes, and granules. Cell..

[148] Liu B, Gao C (2018). Regulation of MAVS activation through post-translational modifications. Curr Opin Immunol.

[149] Chernova TA, Chernoff YO, Wilkinson KD (2017). Prion-based memory of heat stress in yeast. Prion..

[150] Si K, Choi YB, White-Grindley E, Majumdar A, Kandel ER (2010). Aplysia CPEB can form prion-like multimers in sensory neurons that contribute to long-term facilitation. Cell..

[151] Fioriti L, Myers C, Huang YY, Li X, Stephan JS, Trifilieff P, Colnaghi L, Kosmidis S, Drisaldi B, Pavlopoulos E (2015). The persistence of hippocampal-based memory requires protein synthesis mediated by the prion-like protein CPEB3. Neuron..

[152] Tyedmers J, Madariaga ML, Lindquist S (2008). Prion switching in response to environmental stress. PLoS Biol.

[153] Halfmann R, Alberti S, Lindquist S (2010). Prions, protein homeostasis, and phenotypic diversity. Trends Cell Biol.

[154] Lancaster AK, Bardill JP, True HL, Masel J (2010). The spontaneous appearance rate of the yeast prion [PSI+] and its implications for the evolution of the evolvability properties of the [PSI+] system. Genetics..

[155] March ZM, King OD, Shorter J (1647). Prion-like domains as epigenetic regulators, scaffolds for subcellular organization, and drivers of neurodegenerative disease. Brain Res.

[156] Newby GA, Lindquist S (2017). Pioneer cells established by the [SWI+] prion can promote dispersal and out-crossing in yeast. PLoS Biol.

[157] True HL, Lindquist SL (2000). A yeast prion provides a mechanism for genetic variation and phenotypic diversity. Nature..

[158] Griswold CK, Masel J (2009). Complex adaptations can drive the evolution of the capacitor [PSI], even with realistic rates of yeast sex. PLoS Genet.

[159] True HL, Berlin I, Lindquist SL (2004). Epigenetic regulation of translation reveals hidden genetic variation to produce complex traits. Nature..

[160] Newby GA, Lindquist S (2013). Blessings in disguise: biological benefits of prion-like mechanisms. Trends Cell Biol.

[161] Jarosz DF, Lancaster AK, Brown JCS, Lindquist S (2014). An evolutionarily conserved prion-like element converts wild fungi from metabolic specialists to generalists. Cell..

[162] Goncharoff DK, Du Z, Li L. A brief overview of the Swi1 prion-[SWI+]. FEMS Yeast Res 2018;18(6). doi: 10.1093/femsyr/foy061. 10.1093/femsyr/foy061 PMC6001882 29905794

[163] Fleming E, Yuan AH, Heller DM, Hochschild A (2019). A bacteria-based genetic assay detects prion formation. Proc Natl Acad Sci U S A.

[164] Khalil AS, Collins JJ (2010). Synthetic biology: applications come of age. Nat Rev Genet..

[165] Bashor CJ, Collins JJ (2018). Understanding biological regulation through synthetic biology. Annu Rev Biophys.

[166] Purnick PE, Weiss R (2009). The second wave of synthetic biology: from modules to systems. Nat Rev Mol Cell Biol..

[167] Brophy JA, Voigt CA (2014). Principles of genetic circuit design. Nat Methods.

[168] Nandagopal N, Elowitz MB (2011). Synthetic biology: integrated gene circuits. Science..

[169] Cameron DE, Bashor CJ, Collins JJ (2014). A brief history of synthetic biology. Nat Rev Microbiol.

[170] Nielsen AA, Der BS, Shin J, Vaidyanathan P, Paralanov V, Strychalski EA, Ross D, Densmore D, Voigt CA (2016). Genetic circuit design automation. Science.

[171] Yang J, Yan R, Roy A, Xu D, Poisson J, Zhang Y (2015). The I-TASSER suite: protein structure and function prediction. Nat Methods.

[172] Xu D, Zhang Y (2012). Ab initio protein structure assembly using continuous structure fragments and optimized knowledge-based force field. Proteins..

[173] Xia X (2018). Bioinformatics and the cell: modern computational approaches in genomics, proteomics and transcriptomics.

[174] Heffernan R, Paliwal K, Lyons J, Dehzangi A, Sharma A, Wang J, Sattar A, Yang Y, Zhou Y (2015). Improving prediction of secondary structure, local backbone angles, and solvent accessible surface area of proteins by iterative deep learning. Sci Rep.

[175] Xue B, Dunbrack RL, Williams RW, Dunker AK, Uversky VN (2010). PONDR-FIT: a meta-predictor of intrinsically disordered amino acids. Biochim Biophys Acta.

[176] Tartaglia GG, Pawar AP, Campioni S, Dobson CM, Chiti F, Vendruscolo M (2008). Prediction of aggregation-prone regions in structured proteins. J Mol Biol.

[177] Afsar Minhas FUA, Ross ED, Ben-Hur A (2017). Amino acid composition predicts prion activity. PLoS Comput Biol.

[178] Vernon RM, Chong PA, Tsang B, Kim TH, Bah A, Farber P, Lin H, Forman-Kay JD. Pi-pi contacts are an overlooked protein feature relevant to phase separation. eLife. 2018;7:e31486. 10.7554/eLife.31486. 10.7554/eLife.31486 PMC5847340 29424691

[179] Bolognesi B, Lorenzo Gotor N, Dhar R, Cirillo D, Baldrighi M, Tartaglia GG, Lehner B (2016). A concentration-dependent liquid phase separation can cause toxicity upon increased protein expression. Cell Rep.

[180] Sondheimer N, Lindquist S (2000). Rnq1: an epigenetic modifier of protein function in yeast. Mol Cell.

[181] Du Z (2011). The complexity and implications of yeast prion domains. Prion..

[182] Quiroz FG, Chilkoti A (2015). Sequence heuristics to encode phase behaviour in intrinsically disordered protein polymers. Nat Materials.

[183] Simon JR, Carroll NJ, Rubinstein M, Chilkoti A, Lopez GP (2017). Programming molecular self-assembly of intrinsically disordered proteins containing sequences of low complexity. Nat Chem.

[184] Boeynaems S, Holehouse AS, Weinhardt V, Kovacs D, Van Lindt J, Larabell C, Van Den Bosch L, Das R, Tompa PS, Pappu RV (2019). Spontaneous driving forces give rise to protein-RNA condensates with coexisting phases and complex material properties. Proc Natl Acad Sci U S A.

[185] Kato M, Han TW, Xie S, Shi K, Du X, Wu LC, Mirzaei H, Goldsmith EJ, Longgood J, Pei J (2012). Cell-free formation of RNA granules: low complexity sequence domains form dynamic fibers within hydrogels. Cell..

[186] Toombs JA, Petri M, Paul KR, Kan GY, Ben-Hur A, Ross ED (2012). De novo design of synthetic prion domains. Proc Natl Acad Sci U S A.

[187] Taglialegna A, Lasa I, Valle J (2016). Amyloid structures as biofilm matrix scaffolds. J Bacteriol.

[188] Glass DS, Riedel-Kruse IH (2018). A synthetic bacterial cell-cell adhesion toolbox for programming multicellular morphologies and patterns. Cell..

[189] Zhong C, Gurry T, Cheng AA, Downey J, Deng Z, Stultz CM, Lu TK (2014). Strong underwater adhesives made by self-assembling multi-protein nanofibres. Nat Nanotechnol.

[190] Seker UO, Chen AY, Citorik RJ, Lu TK (2017). Synthetic biogenesis of bacterial amyloid nanomaterials with tunable inorganic-organic interfaces and electrical conductivity. ACS Synthetic Biol..

[191] Khalil AS, Lu TK, Bashor CJ, Ramirez CL, Pyenson NC, Joung JK, Collins JJ (2012). A synthetic biology framework for programming eukaryotic transcription functions. Cell..

[192] Bashor CJ, Patel N, Choubey S, Beyzavi A, Kondev J, Collins JJ, Khalil AS (2019). Complex signal processing in synthetic gene circuits using cooperative regulatory assemblies. Science..

[193] Keung AJ, Bashor CJ, Kiriakov S, Collins JJ, Khalil AS (2014). Using targeted chromatin regulators to engineer combinatorial and spatial transcriptional regulation. Cell..

[194] Elowitz MB, Leibler S (2000). A synthetic oscillatory network of transcriptional regulators. Nature..

[195] Stricker J, Cookson S, Bennett MR, Mather WH, Tsimring LS, Hasty J (2008). A fast, robust and tunable synthetic gene oscillator. Nature..

[196] Gaber R, Lebar T, Majerle A, Ster B, Dobnikar A, Bencina M, Jerala R (2014). Designable DNA-binding domains enable construction of logic circuits in mammalian cells. Nat Chem Biol.

[197] Chavez A, Scheiman J, Vora S, Pruitt BW, Tuttle M, Pruitt BW, Lin S, Kiani S, Guzman CD, Wiegand DJ (2015). Highly efficient Cas9-mediated transcriptional programming. Nat Methods.

[198] Gersbach CA, Perez-Pinera P (2014). Activating human genes with zinc finger proteins, transcription activator-like effectors and CRISPR/Cas9 for gene therapy and regenerative medicine. Expert Opin Therapeutic Targets.

[199] Piatek A, Mahfouz MM (2017). Targeted genome regulation via synthetic programmable transcriptional regulators. Crit Rev Biotechnol.

[200] Gaj T, Gersbach CA, Barbas CF (2013). ZFN, TALEN, and CRISPR/Cas-based methods for genome engineering. Trends Biotechnol.

[201] Matsuura S, Ono H, Kawasaki S, Kuang Y, Fujita Y, Saito H (2018). Synthetic RNA-based logic computation in mammalian cells. Nat Commun.

[202] Leisner M, Bleris L, Lohmueller J, Xie Z, Benenson Y (2012). MicroRNA circuits for transcriptional logic. Methods Mol Biol.

[203] Nissim L, Wu MR, Pery E, Binder-Nissim A, Suzuki HI, Stupp D, Wehrspaun C, Tabach Y, Sharp PA, Lu TK (2017). Synthetic RNA-based immunomodulatory gene circuits for cancer immunotherapy. Cell..

[204] Wroblewska L, Kitada T, Endo K, Siciliano V, Stillo B, Saito H, Weiss R (2015). Mammalian synthetic circuits with RNA binding proteins for RNA-only delivery. Nat Biotechnol.

[205] Grunberg R, Serrano L (2010). Strategies for protein synthetic biology. Nucleic Acids Res.

[206] Daringer NM, Dudek RM, Schwarz KA, Leonard JN (2014). Modular extracellular sensor architecture for engineering mammalian cell-based devices. ACS Synthetic Biol..

[207] Bashor CJ, Helman NC, Yan S, Lim WA (2008). Using engineered scaffold interactions to reshape MAP kinase pathway signaling dynamics. Science..

[208] Thompson KE, Bashor CJ, Lim WA, Keating AE (2012). SYNZIP protein interaction toolbox: in vitro and in vivo specifications of heterospecific coiled-coil interaction domains. ACS Synthetic Biol.

[209] Chen Z, Boyken SE, Jia M, Busch F, Flores-Solis D, Bick MJ, Lu P, VanAernum ZL, Sahasrabuddhe A, Langan RA (2019). Programmable design of orthogonal protein heterodimers. Nature..

[210] Gao XJ, Chong LS, Kim MS, Elowitz MB (2018). Programmable protein circuits in living cells. Science..

[211] Cella F, Wroblewska L, Weiss R, Siciliano V (2018). Engineering protein-protein devices for multilayered regulation of mRNA translation using orthogonal proteases in mammalian cells. Nat Commun.

[212] Gordley RM, Williams RE, Bashor CJ, Toettcher JE, Yan S, Lim WA (2016). Engineering dynamical control of cell fate switching using synthetic phospho-regulons. Proc Natl Acad Sci U S A.

[213] Ferreon JC, Jain A, Choi KJ, Tsoi PS, MacKenzie KR, Jung SY, Ferreon AC (2018). Acetylation disfavors tau phase separation. Int J Mol Sci.

[214] Boulay G, Sandoval GJ, Riggi N, Iyer S, Buisson R, Naigles B, Awad ME, Rengarajan S, Volorio A, McBride MJ (2017). Cancer-specific retargeting of BAF complexes by a prion-like domain. Cell..

[215] Monahan Z, Ryan VH, Janke AM, Burke KA, Rhoads SN, Zerze GH, O'Meally R, Dignon GL, Conicella AE, Zheng W (2017). Phosphorylation of the FUS low-complexity domain disrupts phase separation, aggregation, and toxicity. EMBO J.

[216] Nott TJ, Petsalaki E, Farber P, Jervis D, Fussner E, Plochowietz A, Craggs TD, Bazett-Jones DP, Pawson T, Forman-Kay JD (2015). Phase transition of a disordered nuage protein generates environmentally responsive membraneless organelles. Mol Cell.

[217] Drisaldi B, Colnaghi L, Fioriti L, Rao N, Myers C, Snyder AM, Metzger DJ, Tarasoff J, Konstantinov E, Fraser PE (2015). SUMOylation is an inhibitory constraint that regulates the prion-like aggregation and activity of CPEB3. Cell Rep.

[218] Giessen TW, Silver PA (2017). Engineering carbon fixation with artificial protein organelles. Curr Opin Biotechnol.

[219] Shin Y, Berry J, Pannucci N, Haataja MP, Toettcher JE, Brangwynne CP (2017). Spatiotemporal control of intracellular phase transitions using light-activated optoDroplets. Cell..

[220] Reinkemeier CD, Girona GE, Lemke EA (2019). Designer membraneless organelles enable codon reassignment of selected mRNAs in eukaryotes. Science.

[221] Giessen TW (2016). Encapsulins: microbial nanocompartments with applications in biomedicine, nanobiotechnology and materials science. Curr Opin Chem Biol.

[222] Tamura A, Fukutani Y, Takami T, Fujii M, Nakaguchi Y, Murakami Y, Noguchi K, Yohda M, Odaka M (2015). Packaging guest proteins into the encapsulin nanocompartment from Rhodococcus erythropolis N771. Biotechnol Bioeng.

[223] Patterson DP, Schwarz B, Waters RS, Gedeon T, Douglas T (2014). Encapsulation of an enzyme cascade within the bacteriophage P22 virus-like particle. ACS Chem Biol.

[224] Gardner TS, Cantor CR, Collins JJ (2000). Construction of a genetic toggle switch in Escherichia coli. Nature..

[225] Inniss MC, Silver PA (2013). Building synthetic memory. Curr Biol.

[226] Purcell O, Lu TK (2014). Synthetic analog and digital circuits for cellular computation and memory. Curr Opin Biotechnol.

[227] Ajo-Franklin CM, Drubin DA, Eskin JA, Gee EP, Landgraf D, Phillips I, Silver PA (2007). Rational design of memory in eukaryotic cells. Genes Dev.

[228] Burrill DR, Inniss MC, Boyle PM, Silver PA (2012). Synthetic memory circuits for tracking human cell fate. Genes Dev.

[229] Park M, Patel N, Keung AJ, Khalil AS (2019). Engineering epigenetic regulation using synthetic read-write modules. Cell..

[230] Ham TS, Lee SK, Keasling JD, Arkin AP (2008). Design and construction of a double inversion recombination switch for heritable sequential genetic memory. PLoS One.

[231] Farzadfard F, Lu TK (2014). Synthetic biology. Genomically encoded analog memory with precise in vivo DNA writing in living cell populations. Science.

[232] Siuti P, Yazbek J, Lu TK (2013). Synthetic circuits integrating logic and memory in living cells. Nat Biotechnol.

[233] Tang W, Liu DR (2018). Rewritable multi-event analog recording in bacterial and mammalian cells. Science.

[234] Bonnet J, Subsoontorn P, Endy D (2012). Rewritable digital data storage in live cells via engineered control of recombination directionality. Proc Natl Acad Sci U S A.

[235] Kellershohn N, Laurent M (2001). Prion diseases: dynamics of the infection and properties of the bistable transition. Biophys J.

[236] Wang L, Walker BL, Iannaccone S, Bhatt D, Kennedy PJ, Tse WT (2009). Bistable switches control memory and plasticity in cellular differentiation. Proc Natl Acad Sci U S A.

[237] Esvelt KM, Smidler AL, Catteruccia F, Church GM. Concerning RNA-guided gene drives for the alteration of wild populations. eLife. 2014;3:e03401. 10.7554/eLife.03401. 10.7554/eLife.03401 PMC4117217 25035423

